# Morphological and Morphometrical Study of the Human Ossicular Chain: A Review of the Literature and a Meta-Analysis of Experience Over 50 Years

**DOI:** 10.14740/jocmr2369w

**Published:** 2015-12-28

**Authors:** George Noussios, Pantelis Chouridis, Lazaros Kostretzis, Konstantinos Natsis

**Affiliations:** a Laboratory of Anatomy in the Department of Physical Education and Sports Medicine (at Serres) of Aristotle University of Thessaloniki, Thessaloniki, Greece; b ENT Department of Hippokration General Hospital of Thessaloniki, Thessaloniki, Greece; c Department of Anatomy of Medical School, Aristotle University of Thessaloniki, Thessaloniki, Greece

**Keywords:** Ossicles, Incus, Malleus, Stapes, Morphometry, Morphology, Race

## Abstract

The ossicular chain has been known for 500 years and yet there are a small number of morphometrical studies. We reviewed the whole literature that is available online regarding the human ossicular chain from an anatomist perspective and correlated the data from all the papers that showed any relevance. Inclusion and exclusion criteria were developed *a priori*. A thorough description of all ossicular differences has been made and we present their variations in dimensions trying to associate measurements obtained with race. This research included papers spreading on a horizon of over 50 years of worldwide experience. Statistical analysis revealed that there is a great difference in measurements and the results cannot be sufficiently associated. The explanation of this variation in the measurements obtained might be due to errors in the procedure. We conclude that ossicular chain reveals a great variety, and propose that a measurement protocol for auditory ossicles must be widely performed.

## Introduction

**Figure 1:**
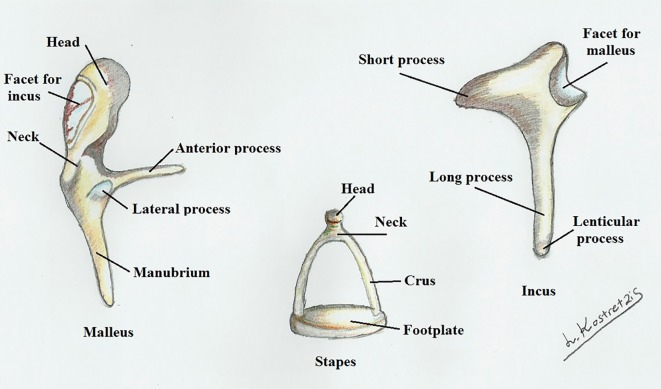
Malleus, incus and stapes.

**Figure 2:**
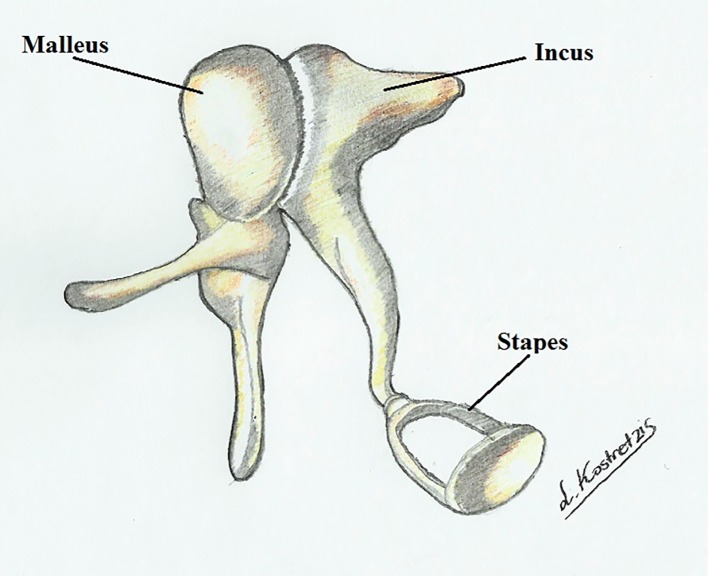
Articulation of the auditory ossicles.

The ossicular chain consists of a complex of bones, which are ingrained deep inside the temporal bone in the tympanic cavity (Fig. 1). Considering the multiple connections between the articulated ossicles (Fig. 2), this area is characterized by considerable anatomic variations as well as a plethora of congenital disorders.

These alterations create essential issues in the gradual and smooth transmission of the sonic waves from the tympanic membrane and amplify them towards the surface of the oval window. The thorough knowledge of these divergences and the morphometrical aberrations is of great importance in the surgical procedures considering the middle ear.

## Literature Search Methods

In our study, two reviewers (P.C. and L.K.) searched Pubmed, Scopus and Cochrane library using the terms ossicles, malleus, stapes, and incus, and selected only the papers regarding studies on humans with morphological or morphometrical recordings. We included original articles regarding peer review in the English language published prior to May 2015. The ossicles of the human fetus as we mentioned before gain in mass and dimensions in their full size *in utero* and a postnatal growth and diversification is insignificant [1], thus we included researches that used newborns ossicles in addition to adults. Review articles and case reports were excluded from our research. Using the full text, two senior reviewers (G.N. and K.N.) performed a second screening of the studies and data were extracted for analysis. The main goal of this study was to determine if any substantial difference regarding the aforementioned anatomic recordings among races exists. Our second objective was to summarize the literature and study the homogeneity of measurements in the course of time.

Papers regarding the malleus that are also figurative in morphology are quite few. We chose those papers that analyzed over 40 individual ossicles and had their measurements obtained using stereoscopic microscopes and micrometers. Padmini and Rao [2] evaluated 100 mallei in which the only variation consisted in the lateral process and the cor manubrium (the handle of malleus). This process was thick and broad in four cases and completely absent in two. Six of them had no neck from the caput (head) to manubrium and also long and large lateral process. Mogra et al [3] observed 66 mallei. In this paper, 59 out of 66 had the neck at presence whilst in seven were absent. The curve of the handle was present in 28 and complete absence of it was found in 38. Unur et al [4] performed a thorough investigation in 40 newborns ossicles. In this study, the writers observed a presence of curving of the manubrium in 20 of them and complete absence of the curving in 20. Todd and Creighton [5] observed in 82 mallei an absence of the lateral process in one case and inflected manubrium.

Incus was the most stable of the ossicles. Morphologically, it was studied by Padmini and Unur. By their observations, Padmini in 100 ossicles found out that four had a curved long process while Unur found no curving. Instead Unur observed a notch in the short process in 17 out of 40 ossicles that was not present in Padmini’s data. Todd also found no notch present in the short process and an anterior curving of the long process in the great majority of them.

Regarding stapes morphology all the papers reveal great variations. The only solid data that can be inter-checked are the presence or absence of the foramen of the stapes. Other not so characteristic differences are in the shape of the foramen. Padmini with 100 and Unur with 40 stapes observed a combination of circular, oval, arched, triangular and tunnel shaped foramen as also a highly deviated head of the stapes. They also observed an atresia of foramen in two cases. Wadhwa et al [6] studied 10 stapes in which one had an extremely long neck and obturators foramen of various shapes such as oval, circular, triangular and semicircular.

## Results

The statistical analysis of the papers was made using SPSS program. The statistical value that was evaluated was the weighted mean value of the different dimensions of the ossicles. The below tables show the papers that we analyzed and reveal our conclusions.

Table 1 [4, 6, 7-12] shows the diameters of the stapes from various researchers. Dass et al [7] by far studied the majority of the ossicles. Other reviewers such as Harneja and Chaturvedi [8], Arensburg et al [9], Wadhwa et al [6] and Rathava et al [10] obtained data from almost any value that is available in the stapes superstructure whilst Awengen et al [11] and Farahani and Nooranipour [12] measured only the width of the footplate.

**Table 1:** Data of Stapes Diameters

	Values, mean ± SD (min - max)							
	Unur et al, 2002 [4]	Dass et al, 1966 [7]	Harneja and Chaturvedi, 1973 [8]	Arensburg et al, 1981 [9]	Wadha et al, 2005 [6]	Rathava et al, 2013 [10]	Awengen et al, 1995 [11]	Farahani and Nooranipour, 1995 [12]
Total height	3.22 ± 0.31	3.29 (2.80 - 3.93)	3.12 ± 0.21 (2.50 - 3.50)	3.20 ± 0.21 (2.89 - 3.72) (n = 19)	3.41 ± 0.20 (3.06 - 3.71)	3.33 ± 0.25 (2.86 - 3.90)	-	-
Length of basis stapedis	2.57 ± 0.33	2.79 (2.29 - 3.30)	2.68 ± 0.27 (1.75 - 3.25)	2.8 ± 0.15 (2.49 - 3.05)	2.97 ± 0.31 (2.64 - 3.56)	2.78 ± 0.15 (2.41 - 3.11)	-	-
Width of basis stapedis	1.29 ± 0.22	1.43 (0.42 - 19.94)	1.26 ± 0.08 (1.10 - 1.50)	1.3 ± 0.07 (1.23 - 1.45)	0.39 ± 0.10 (0.19 - 0.56)	1.34 ± 0.13 (1.05 - 1.73)	2.48 (2.06 - 2.98)	2.298 (1.928 - 3.050)
N	40	165	48	18	10	60	10	12

**Figure 3:**
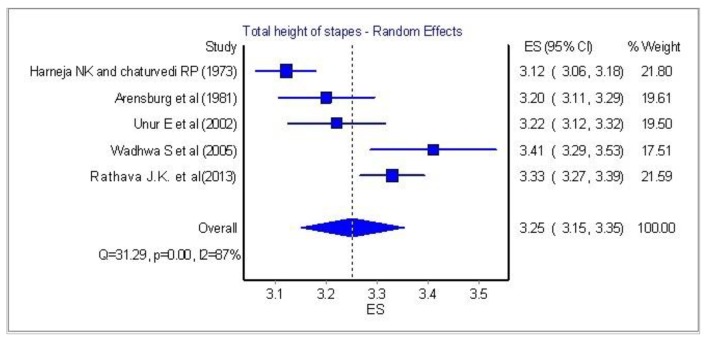
Total height of stapes: statistical analysis of five papers.

Statistical analysis in total height of the stapes was performed using the above five papers in Figure 3 (the most recent of them was the paper of Rathava et al [10], since they provided significant data that were able to be analyzed).

**Figure 4:**
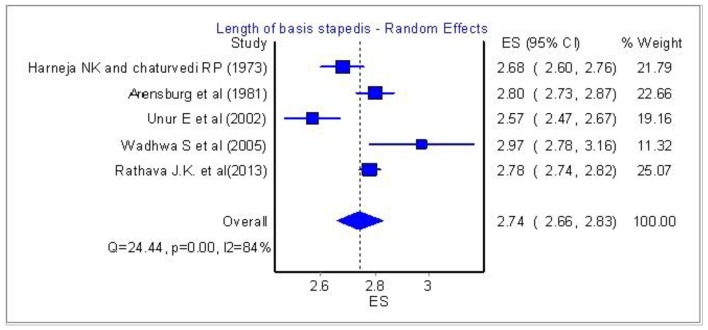
Length of basis stapedis: Analysis of five papers.

**Figure 5:**
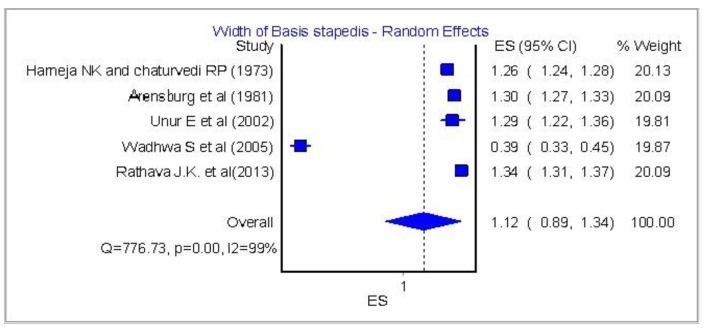
Width of basis stapedis: analysis of five papers.

Figures 4 and 5 show a significant homogeneity in four papers and only the research of Wadhwa et al [6] is quite variable.

**Figure 6:**
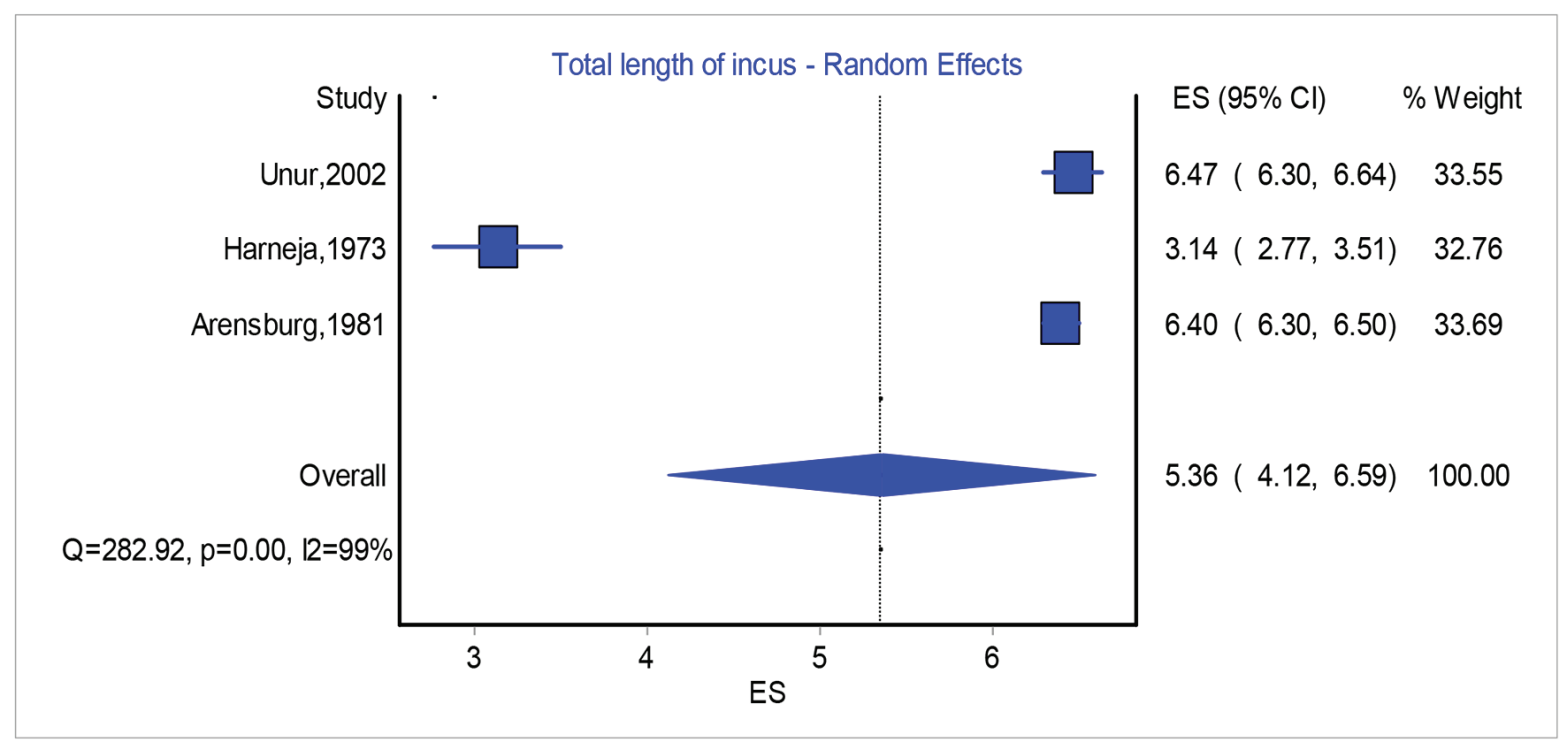
Total length of incus: analysis of three papers.

Regarding incus, the papers that measure dimensions with great accuracy are quite few. The values that could be statistically used and compared were three: Unur et al [4], Harneja and Chaturvedi [8] and Arensburg et al [9] (Table 2 and Fig. 6) [4, 8, 9, 13-15]. Papers from Chien et al [13], Toth et al [14] and Kwok et al [15] were excluded from the analysis due to lack of adequate measurements.

**Table 2:** Incus Diameters

	Values, mean ± SD (min - max)					
	Unur et al, 2002 [4]	Chien et al, 2009 [13]	Harneja and Chaturvedi, 1973 [8]	Toth et al, 2013 [14]	Kwok et al, 2006 [15]	Arensburg et al, 1981 [9]
Total length	6.47 ± 0.55	-	3.14 ± 0.19 (2.80 - 3.75) (SE)	-	-	6.4 ± 0.24 (6.04 - 6.93)
Total width	4.88 ± 0.47	-	1.82 ± 0.14 (1.60 - 2.25) (SE)	-	-	
Distance between processes’ tips	6.12 ± 0.43	-	-	-	-	
Long process diameter at 1.5 mm from tip (prothesis attachment)	-	0.630 (0.260 - 1.10) ± 0.134	-	0.52 - 1.15	0.66 ± 0.05 - 0.81 ± 0.1	
N	40	103	50	50	11	22

The final ossicle that we studied was the one with the most papers and respectfully had a lot of variables. The malleus is the largest of the ossicular chain and by far the most visible through the tympanic membrane in clinical examination. Papers from Singh et al [16], Unur et al [4] and Mogra et al [3] have a plethora of values. On the other hand, Vinayachandra et al [17], Harneja and Chaturvedi [8] and Arensburg et al [9] measured a part of the available values.

**Figure 7:**
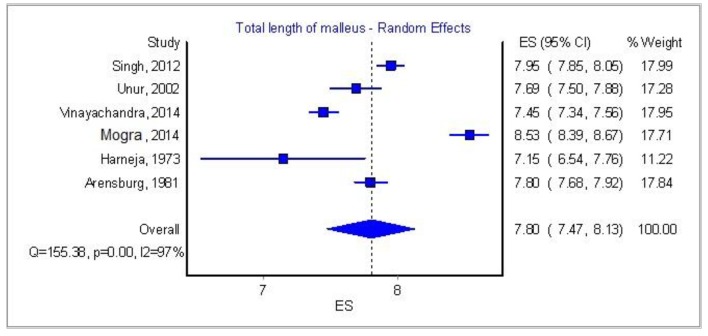
Total length of malleus: analysis of six papers.

**Figure 8:**
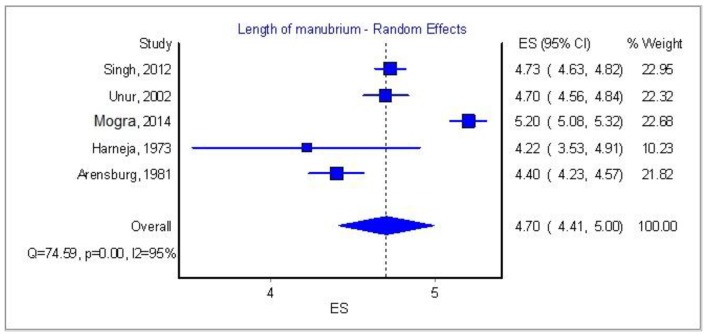
Length of manubrium of malleus: analysis of five papers.

**Figure 9:**
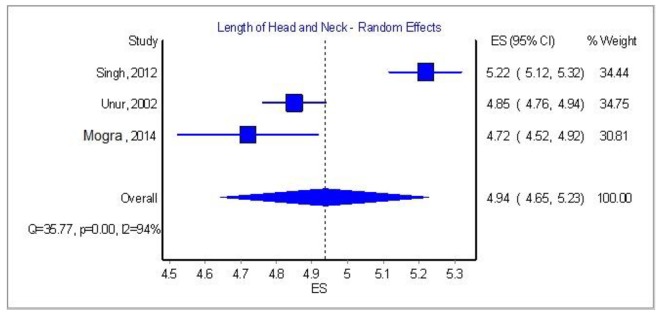
Length of head and neck of malleus: analysis of three papers.

Table 3 and Figures 7-9 [3, 4, 8, 9, 16, 17] show some important features of the previous studies. We tried to correlate the race of the specimens with the mean dimensions of all the ossicles but the differences between the races were of no importance. One paper from South Africa by Oschman and Meiring [18] claimed that there is a statistically significant difference amidst the Caucasian and Negroid race, but it was not possible to account it in our meta-analysis since the authors did not provide a standard error or standard deviation, thus this paper was excluded from the race research.

**Table 3:** Malleus Diameters

	Value, mean ± SD (min - max)						
	Singh et al, 2012 [16] - right	Singh et al, 2012 [16] - left	Unur et al, 2002 [4]	Vinayachandra et al, 2014 [17]	Mogra et al, 2014 [3]	Harneja and Chaturvedi, 1973 [8]	Arensburg et al, 1981 [9]
Total length	7.947 ± 0.415	7.9467 ± 0.401	7.69 ± 0.60	7.45 ± 0.39 (6.94 - 7.78)	8.53 ± 0.58	7.15 ± 0.31 (SE) (6.60 - 8.00)	7.8 ± 0.35 (7.01 - 8.41) (n = 31)
Length of manubrium	4.762 ± 0.45139	4.726 ± 0.376	4.70 ± 0.45		5.20 ± 0.48	4.22 ± 0.35 (SE) (3.75 - 5.20)	4.4 ± 0.47 (3.56 - 5.65) (n = 30)
Length of head and neck	5.237 ± 0.3409	5.2172 ± 0.400	4.85 ± 0.29		4.72 ± 0.82		
N	60	60	40	50	66	50	

## Discussion

The embryological origin of the ossicles is well studied in the previous decades. As it has been demonstrated with great detail for almost a century, the cartilage of the second pharyngeal arch (hyoid arch) is placed opposite to the first pharyngeal pouch and it is dorsally deviated the pharyngotympanic tube. The first pharyngeal (or branchial) arch, also called mandibular arch is positioned just in front of the first pouch. During 4 - 6 weeks of embryonic development, the proximal edges of the cartilages start to form the ossicles of the middle ear. The head of the malleus, the body and short crus of the incus derive from Meckel’s cartilage whilst the manubrium of the malleus, the long crus and body of incus as also the superstructure of stapes (head, neck, anterior and posterior crus additional to the tympanic surface of the footplate) are formed from Reichert’s cartilage. The labyrinthus (or vestibular) surface of the base along with its annular ligament, a small fibrous, ring, is being formed by the otic capsule, a mesenchymal shell structuring the inner ear. Stapes at its origin has a ring-like scheme and surrounding its artery. At about 8.5 weeks, the incudostapedial joint arises, articulating the ossicles and containing a plenteous amount of elastic fibers. The initial mesenchymal structures are being replaced by cartilaginous models and growth furthermore to adult’s size at about 15th week. An earlier report [19] confirmed that ossification of the cartilages starts at the 15th week for malleus and incus and 18th week for stapes, finalizing the bone structure at about 25th week.

The ear ossicles seem to have firstly been described in the 16th century. An earlier report [20] stated that Vesalius described incus and malleus in 1543 in his monumental work “De humani corporis fabrica” (the fabric of the human body), while Eustachius and especially Ingrassia seems to take the credit for first depicting the stapes in 1546 [21]. Eustachi Bartolomeo granted to be the founder of Descriptive Anatomy. The Greek Alcmaion (5th century BC), anatomist, philosopher and apprentice of Pythagoras have written the acclaimed first Medical book “Concerning Nature” in which fragments have been saved and have described the eustachian tube and perhaps the “traversing by a chain of small bones” of the middle ear whilst others consider that he believed that the external sound is picked up by empty space in the inner ear [22].

The occurrence of all congenital malformations of the ear varies between 1/10,000 and 1/15,000 as described by Wetmore et al [23]. There is rather quite substantial amount of ossicular deformities for such a limited space area. Alterations can happen unilateral or bilateral. When the latter is being observed, most commonly an autosomal dominant prevalence is present (25-40%). Todd and Creighton [5] suggest that the embryological formations of the second branchial arch have more changeability than the first arch ones.

At first, incus is usually affected with hypoplasia or complete aplasia in combination with hypoplasia of malleus. Occasionally the anomaly can happen individually only in incus. It is liable to fixation in the epitympanum. Congenital absence of the long process usually associates with the aplasia of the stapes and aplasia of the handle of the malleus, supporting their common embryological origin.

The most common solitary abnormality of all ossicles is the fixation of stapes’ footplate (ankylosis), contributing to all congenital anomalies of the middle ear at about 40%. The predominant theory of this anomaly is the ossification of the peripheral mesenchyme of the footplate. Solid aplasia of the stapes is very rare whilst many forms of hypoplasia have been described such as a small or absent crura, a blob-like structure, etc. In addition, the incudostapedial disconnection is a very characteristic variation of the articulated ossicles, although it is usually due to traumatic factors [24].

Malleus has its segment in the plethora of anatomical variations of the ossicles. One of the commonest and quite controversial is the congenital bony fixation [25]. Complete absence or hypoplasia is not so usual and is combined with agenesis or hypoplasia of the other ossicles. Earlier studies by Sando et al [26] revealed that congenital abnormalities of the ossicles accompany the malformations of the facial nerve and its aberrant development in the cranium.

Our research included papers spreading on a horizon of over 50 years of worldwide experience.

The papers that provided adequate data for race comparison originated mostly from India by researchers such as Dass et al (1966) [7], Harneja and Chaturvedi (1973) [8], Arensburg et al (1981) [9], Wadha et al (2005) [6], Singh et al (2012) [16], Padmini and Rao (2013) [2], Rathava et al (2013) [10], Mogra et al (2014) [3] and Vinayachandra et al (2014) [17], and single papers from Turkey (Unur et al, 2002) [4], USA (Chien et al, 2009) [13], Austria (Toth et al, 2013) [14], Australia (Farahani and Nooranipour, 2008) [12] as well as two papers from Germany (Awengen et al, 1995 [11] and Kwok et al, 2006 [15]). Our assumption that there is an ossicular difference among the races was of non-important significance. We tried to correlate the chronological age of the papers assuming that the earlier the research was contacted the vaguer the calculations, but none of this hypothesis was of any basis.

The statistical analysis revealed that there is a great difference in measurements and the results cannot be sufficiently associated. The explanation of this variation in the measurements obtained (in some researches in the same race and also region) might be due to errors in the procedure.

Thus we propose that a measurement protocol for auditory ossicles should be widely accepted and practiced. From the literature the most solid set of measurements was defined by Quam and Rak [27]. According to them, the researchers primarily must determine the X and Y axes of the ossicles and then all the distances and angles should be inscribed. The most efficient and accurate method seems to be the use of computed tomography that can directly measure three-dimensional objects as stated by Hallgrimsson et al [28]. Another efficient method is the projection of an ossicle in a photographic print or digitally on a screen of a microscope and then the measurements can be obtained. The later method is more easily carried out, but entrails errors regarding the projection of such small objects into a screen (parallax error). A study by Flohr et al [29] providing measurements from two different technicians showed an inter-observer error ranging from 2% to 2.63%, hence there must be a minimum of at least two measurements of the same value for each separate ossicle. The present study has revealed that papers from previous years, despite measuring common dimensions, cannot be sufficiently interpreted in terms of mean weighted value of measurements or race. The above methods might be an answer in performing a more well-organized and error-minimum standardized procedure.

## Conclusions

Auditory ossicles are under examination since their discovery. Their contribution in the hearing process postures them in the forefront of hearing problems researches. The ossicular chain has shown great variety and complexity in measurements as well as in morphology taken from all researchers. Statistical analysis performed by the authors revealed that there is great diversity in their comparative values and a new standard method must be accomplished in order to achieve much more accurate measurements.
